# The Comparative Evaluation of the Effects of Tongue Cleaning on Existing Plaque Levels in Children

**DOI:** 10.5005/jp-journals-10005-1216

**Published:** 2013-10-14

**Authors:** J Jasmin Winnier, S Rupesh, Ullal Anand Nayak, Venugopal Reddy, Arun Prasad Rao

**Affiliations:** Associate Professor, Department of Pediatric and Preventive Dentistry Dr DY Patil Dental College and Hospital, Navi Mumbai, Maharashtra, India; Associate Professor, Department of Pedodontics and Preventive Dentistry, Pushpagiri College of Dental Sciences, Kottayam, Kerala India, e-mail: yourpediatricdentist@gmail.com; Professor, Department of Pedodontics and Preventive Dentistry Mahatma Gandhi Dental College, Jaipur, Rajasthan, India; Professor, Department of Pediatric and Preventive Dentistry, Rajah Muthiah Dental College and Hospital, Annamalainagar, Tamil Nadu India; Professor, Department of Pediatric and Preventive Dentistry, Rajah Muthiah Dental College and Hospital, Annamalainagar, Tamil Nadu India

**Keywords:** Tongue cleaning, Tongue scraping, Tongue brushing, Tooth brushing, Existing plaque

## Abstract

The present study compared and evaluated the effects of tongue scraping and tongue brushing on existing plaque levels in children. The investigation was a single blind, stratified comparison of three parallel groups of children who performed either tongue scraping or tongue brushing along with tooth brushing or only tooth brushing twice daily under professional supervision for a 21 day period. Dental plaque was recorded using the plaque index described by Silness and Loe at baseline, on day 10 and on day 21. All data was subjected to statistical analysis using Wilcoxon's Signed Ranks Sum Test and Mann-Whitney U-test. The results of the present study show that the tongue scraping and tongue brushing groups showed statistically significant reductions in plaque levels after 10 days and also after 21 days. It was also noted that both tongue scraping and tongue brushing were equally effective in reducing the plaque load in children.

**How to cite this article:** Winnier JJ, Rupesh S, Nayak UA, Reddy V, Rao AP. The Comparative Evaluation of the Effects of Tongue Cleaning on Existing Plaque Levels in Children. Int J Clin Pediatr Dent 2013;6(3):188-192.

## INTRODUCTION

Dental caries is undoubtedly a multifactorial disease and dental plaque plays a major role in its pathogenesis. Dental plaque is a biofilm, it is formed by colonizing bacteria trying to attach itself to the smooth surface of the tooth. It is considered to be a complex, metabolically interconnected, highly organized bacterial system consisting of dense masses of microorganisms embedded in an intermicrobial matrix. The pellicle, which is an organic bacteria-free film, deposits on the tooth surfaces within nanoseconds after vigorous tooth brushing or polishing. Then, the bacteria start to colonize the tooth surface. The dental plaque, in sufficient concentration, can disturb the host-parasite relationship and cause dental caries. The plaque thickness differs depending on the locally prevailing oral cleansing forces, oral hygiene and other factors such as salivary components.

The oral surfaces are colonized by over 500 bacterial species and tongue has the largest bacterial load of any oral tissue and makes the greatest contribution to the bacteria found in the oral cavity.^1^ More than 100 bacteria may be attached to a single epithelial cell on top of the tongue, whereas only about 25 bacteria are attached to each cell in other areas of the oral cavity.^2^ There is also a continuous shedding of cells of the surface layer of epithelium from the tongue and palate and the availability of oral debris from these sites could contribute to plaque formation on the teeth.^3^

Therefore reducing the load of bacteria on the tongue may help in reducing the rate of plaque formation on the tooth.

Tooth brushing is the most frequently practiced oral hygiene procedure, but it cleans only selected areas of the tooth and the gingiva. Even when adjunctive methods of oral hygiene, such as water jet devices, dental floss and toothpicks are successfully employed, the patient is assured only of clean tooth surfaces and gingiva.^4^

Few references appear in the early 20th century dental literature about tongue scraping or cleaning.^5^ The last few years however, have brought forth scores of devices and gadgets to clean the tongue.^6^

The effects of these simple indigenous adjunctive oral hygiene procedures in children have not been documented and there is a relative paucity of studies in the dental literature in this regard. The present study was attempted to compare and evaluate the effects of tongue scraping and tongue brushing on plaque levels in 9 to 12-year-old children.

## MATERIALS AND METHODS

The investigation was a single-blind, stratified comparison of three parallel goups of children who performed either tongue scraping, tongue brushing along with routine tooth brushing or only tooth brushing (Placebo group) twice daily under professional supervision for a 21-day period. All the school children in the age group of 9 to 12 years from a residential school (boarding school) for boys were examined using oral health survey forms and 45 children were selected based on the following selection criteria:

1. Subjects in the age group of 9 to 12 years.
2. At least four restored, decayed and/or missing teeth (DMFS/dmfs > 4).
3. Subjects adhering to twice-daily tooth brushing routine (using toothbrush and nonfluoridated toothpaste) and practicing no other oral hygiene measures, either professional or home based, other than the requisites of the research project.
4. No history of antibiotic usage during the past 1 month.
5. No orthodontic appliance worn.
6. No abscess, draining sinus, cellulitis or other conditions requiring emergency dental treatment.
7. Participant cooperation and acceptance of the treatment regimen.
8. No medical/hereditary condition or long term/ recent/ current regimen of medication that necessitate diet modification.

Verbal consent from children and signed consent forms from parents or guardians were obtained after the nature of the study and the possible risks were fully explained*.* The study project was approved by the concerned ethics committee. The subjects were then divided into three balanced groups of 15 subjects each that were comparable in terms of gender (only male subjects could be chosen as the study was conducted in a boarding school for boys), age, number of teeth, average dmfs/DMFS. The oral hygiene measures assigned to each group were as follows:

*Group I:* This group involves 15 participants constituted the tongue-scraping group. Participants were given a metal tongue scraper and asked to scrape the dorsum of the tongue twice daily. The tongue scraper used in the present study was an inverted V shaped stainless steel scraper with plastic handles at the ends. The scraper was grasped by the handles and the apex of the inverted V made of a flattened stainless steel strip was placed in the dorsum of the tongue. The following instructions were given:

1. Place the tongue as far out of the mouth as possible and place the tongue scraper as far posterior as possible on the tongue (comfortable enough to avoid gagging).
2. Apply force on the scraper to flatten the tongue, making it conform to the surface of the tongue and pull the scraper forward slowly but firmly up to the tip of the tongue.
3. Spit out the excess saliva and/ or debris that accumulate on the tongue and remove the debris from the tongue scraper by placing it under a stream of running water.
4. Repeat the procedure five times.

*Group II:* Involving 15 participants constituted the tongue-brushing group. Participants were given a soft multitufted nylon toothbrush with a minihead and asked to brush the dorsum of the tongue twice daily. The following instructions were given:

- Place the tongue as far out of the mouth as possible. Place the brush as far posterior as possible on the tongue in the midline (comfortable enough to avoid gagging) and give five firm forward and backward strokes (moving the brush till the tip of the tongue and back).
- Spit out the excess saliva and debris that accumulates on the tongue and clean the brush by placing it under a stream of running water.
- 3. Repeat the procedure on either side of the midline of the tongue.

*Group III:* Involving 15 participants who continued with their regular tooth brushing regimen twice daily.

A monitor trained to instruct the subjects and to assist them to perform the various oral hygiene procedures directly supervised the treatment regimen at the residential school hostel where the participants were staying. The procedures were performed twice daily (once in the morning after breakfast and once after the evening meal).

### Clinical Procedure

Clinical assessments were performed at the residential school by a single examiner using portable dental operatories and accepted methods of infection control. The monitor coded the study subjects from 1 to 45 before clinical examination by the examiner to ensure that at no time was the examiner aware of the group assignment of any subject. The data was later decoded at the end of the investigation.

Dental plaque was scored by the examiner on individual plaque assessment forms. Separate plaque assessment forms were used at each examination. The plaque was disclosed using disclosing solution and the examiner performed the clinical measurements at the same time of the day throughout the study. The plaque index described by Silness and Loe (1964) was used to assess the dental plaque in children.^7^The teeth used for plaque index measurements were 16, 12, 24, 36, 32, 44. Deciduous counterparts were used in case of unerupted permanent teeth. Each of the four gingival areas of the tooth was given a score ranging from 0-3. By adding the scores for each tooth and dividing by the number of teeth examined, plaque index for the individual subject was obtained.

### Statistical Evaluation

All the data was entered into a database on Microsoft Excel and analyzed using SPSS software with two way ANOVA (for overall group mean comparisons), Wilcoxon's Signed Ranks Sum Test (for intragroup comparison of differences between baseline, days 10 and 21 examinations of plaque index) and Mann-Whitney U-test (for intergroup comparisons of plaque index).

## RESULTS

The sample characteristics of the study population are presented in Table 1. The mean values of plaque index data of all the groups at baseline, 10 days and after 21 days are shown in Table 2.

**Table 1:** **Table 1:** Sample characteristics of the study population.

*Groups*	*Mean and Standard deviation values*			
	*Age*	*No. of teeth*	*dmft/DMFS*	*dmfs/DMFS*
Group I N = 15	10.50 ± 1.09 (9-12)	23.42 ± 0.9	7.00 ± 2.37	11.50 ± 6.7
Group II N = 15	10.50 ± 1.00 (9-12)	23.42 ± 0.9	6.92 ± 2.88	11.42 ± 7.29
Group III N = 15	10.53 ± 1.16 (9-12)	23.42 ± 0.79	7.67 ± 3.75	11.33 ± 8.04

**Table 2:** **Table 2:** The mean and standard deviation values of plaque index (log values) at baseline, day 10 and day 21 of all groups

*Groups*	*Mean and standard deviation values at*				
	*Baseline*		*Day 10*		*Day 21*
I	1.60 ± 0.49		1.52 ± 0.50		1.46 ± 0.50
II	1.62 ± 0.46		1.54 ± 0.46		1.47 ± 0.45
III	1.53 ± 0.53		1.52 ± 0.51		1.58 ± 0.45

### Intragroup Comparison

Comparison of the differences in plaque index data between baseline and day 10, baseline and day 21 and between days 10 and 21 are presented in Table 3. Children who performed tongue scraping and tongue brushing showed statistically significant reductions in plaque scores after 10 days and also after 21 days. Significant results were also obtained when comparing the results between days 10 and 21. However, children who continued with tooth brushing alone exhibited no significant reductions in plaque scores after 10 days and even after 21 days when compared with the baseline. No significant difference was found between the days 10 and 21.

### Intergroup Comparison

The intergroup comparisons of the plaque index data at baseline, days 10 and 21 are presented in Table 4. No significant difference in plaque scores was observed between groups I, II and III at baseline and after 10 days. After 21 days, groups I and II showed statistically significant differences over group III (p = 0.047 and p = 0.032, respectively). No significant differences were observed between groups I and II (p = 0.977).

## DISCUSSION

Plaque is an important source for salivary mutans streptococci and the tongue provides the largest bacterial load compared to any other oral tissues and makes the greatest contribution to bacteria. In the evaluation of caries risk, it is thus interesting to estimate the effects of mechanical tongue cleaning techniques on plaque.

Age is a critical factor in subject selection for many reasons, of which the most important is the number of tooth surfaces at risk. Subjects with a mean age of approximately 11 years were chosen because they were entering a period of high caries activity, with many permanent teeth erupting.^8^Subjects with either rampant tooth decay or very poor oral hygiene were also included in the study as it was important to determine whether the protocol remained effective for all ranges of hygiene with different levels of exixting plaque. Because our study was conducted in a residential school for boys, female subjects could not be selected. However, epidemiological studies in caries prevalence have not shown any significant difference in caries susceptibility of boys and girls at an average age.^9^

**Table 3:** **Table 3:** Comparison of differences between baseline, day 10 and day 21 examinations for plaque index data using Wilcoxon's Signed Rank Sum Test

*Groups*		*Baseline vs day 10*				*Baseline vs day 21*				*Day 10 vs Day 21*		
		*Z*		*p*		*Z*		*p*		*Z*		*p*
I		2.955		0.003***		3.066		0.002***		2.820		0.005***
II		2.274		0.023**		2.943		0.003***		3.097		0.002***
III		0.393		0.694*		1.338		0.181*		1.582		0.114*

***Highly significant; **Significant; *Not significant

**Table 4:** **Table 4:** Inter-group comparison of plaque index data at baseline, day 10 and day 21 using Mann-Whitney U-test

*Groups*		*Baseline*				*10th day*				*21st day*		
		*Z*		*p*		*Z*		*p*		*Z*		*p*
I *vs* II		0.029		0.977*		0.058		0.954*		0.029		0.977*
I *vs* III		0.694		0.488*		1.041		0.298*		2.059		0.047**
II *vs* III		0.636		0.524*		1.186		0.236*		2.019		0.032**

***Highly significant; **Significant; *Not significant

As the present study was conducted in a residential school, all the subjects consumed the same diet during the period of investigation. Dental caries is a dietary carbohydrate modified infectious disease, because the major causative factors are believed to be local in nature.^10^ The frequency of exposure to a cariogenic diet and the form of intake of cariogenic food substances appear to be important factors in the development of dental plaque and caries.^11^As diet–which is an important factor in dental caries and plaque flora was controlled in our study, the different tongue cleaning procedures were possibly given the best chance of demonstrating their efficacy against dental plaque.

Dental plaque was assessed at baseline, days 10 and 21 using the plaque index of Silnes and Loe (1964).^12^ This index has been criticized as being highly subjective and it is therefore recommended that a single examiner be trained and used with each group of patients throughout the clinical trial.^13^ In our study, the dental plaque measurements were made by a single examiner.

The results of this clinical trial showed that both the tongue scraping and the tongue brushing groups exhibited statistically significant reductions in plaque scores when baseline values were compared with post-treatment values after days 10 and 21. These results showed that there is a definite decrease in the plaque scores even within 10 days of regular tongue cleaning of which tongue scraping shows a slightly more statistically significant result than tongue brushing. The control group, however, did not show any statistically significant change, when the days 10 and 21 values were compared with baseline values.

Intergroup comparisons reveled that there were no statistically significant differences in plaque scores between the three groups at baseline. This implies that all groups were statistically equivalent before the start of treatment. It was observed that after 21 days, both the tongue scraping and tongue brushing groups showed statistically significant differences over the control group.

It has been demonstrated that habitual tongue brushing reduces or eliminates the organisms on the tongue which form plaque *in vitro*^4^ and that numbers and types of plaque organisms are altered by a regimen of daily tongue brushing.^14^ A reduction in plaque formation on teeth when cleaning the tongue has been noted ^15^ and tongue brushing, when supplemented with the most advocated regime of tooth brushing, reduced the initial rate of plaque formation and total plaque accumulation.^16^ Rupesh et al, (2011) evaluated the effects of tongue cleaning in mutans streptococci levels and reported significant reductions in 10 days and after 21 days.^17^ The observations from these studies support the reduction in plaque scores demonstrated by tongue cleaning in the present study.

Though research has shown that toothbrushes are inferior to scraping debridement implements in their ability to remove debris, in the present study, both these procedures were found to be equally effective in reducing plaque levels.

The oral hygiene measures used in our study were simple, could be carried out fast and the benefits for most children far outweighed the small investment and time required to accomplish the procedure. Also none of the participants had any compliance problems, aberrations, lesions, etc. any time during the study after using either the scraper or the brush. The concept of tongue cleaning is so logical and so simple that prevention oriented people should need only minimal encouragement to incorporate tongue cleaning into their oral hygiene routine. Our study was a novel attempt designed to simulate a realistic home regimen in which the subjects either performed tongue scraping or tongue brushing daily while continuing their normal twice-daily tooth brushing routine. In this context, it is noteworthy that the reductions plaque scores in our study occurred in addition to the effects of twice daily tooth brushing.

Thus, in this new era of dentistry, it is important that research prove the need to include the tongue in all oral hygiene measures. With tongue scraping/brushing becoming established as excellent tools for reducing the levels of plaque in the oral cavity, it would be of great interest to compare their efficacy with other more mainstream methods.

## CONCLUSION

The tongue scraping and tongue brushing groups showed statistically significant reductions in plaque counts after 10 days and also after 21 days when performed along with tooth brushing. Thus, in the present study, these simple tongue cleaning procedures emerged as effective adjunctive oral hygiene measures.
